# Effects of dietary cation and anion difference on eating, ruminal function and plasma leptin in goats under tropical condition

**DOI:** 10.5713/ajas.19.0288

**Published:** 2019-08-23

**Authors:** Thiet Nguyen, Somchai Chanpongsang, Narongsak Chaiyabutr, Sumpun Thammacharoen

**Affiliations:** 1 Department of Physiology, Faculty of Veterinary Science, Chulalongkorn University, HenriDunang street, Bangkok 10330, Thailand; 2 Department of Agricultural Technology, Faculty of Rural Development, Cantho University, 3/2 street, Cantho city 94000, Vietnam; 3 Department of Animal husbandry, Faculty of Veterinary Science, Chulalongkorn University, HenriDunang street, Bangkok 10330, Thailand; 4 The Academy of Science, The Royal Society of Thailand, Dusit, Bangkok 10300, Thailand

**Keywords:** Dairy Goat, Dietary Cation and Anion Difference (DCAD), Dry Matter Intake, Leptin, Rumen Fermentation

## Abstract

**Objective:**

This study was carried out to determine the effects of elevated dietary cation and anion difference (DCAD) on dry matter intake (DMI) and ruminal fermentation pattern in lactating dairy goats under tropical conditions.

**Methods:**

Ten dairy goats were divided into two groups of five animals each. The groups received diets at different DCAD levels, either a control diet (22.81 mEq/100 g dry matter [DM], DCAD-23) or a DCAD-39 diet (39.08 mEq/100 g DM, DCAD-39). After parturition, DMI and water intake were recorded daily. Ruminal fluid and urine were collected, and nutrient digestibility measurements were carried out at 8th weeks postpartum (PP-8). Blood samples were collected at PP-4 and PP-8 to measure plasma leptin.

**Results:**

Dry matter intake/body weight (DMI/BW) at PP-8 of the animals fed the DCAD-39 diet was significantly higher than those fed with DCAD-23 diet (p<0.05). Animals fed with DCAD-39 consumed more water than those fed DCAD-23 over 24 h, particularly at night (p<0.05). Ruminal pH, acetate concentration, and urinary allantoin excretion increased with the DCAD-39 diet, whereas ruminal butyrate concentration was lower with the DCAD-39 diet. On the other hand, other ruminal parameters, such as total volatile fatty acid concentration, propionate molar proportion and acetate/propionate average ratio, were not affected by increased DCAD supplementation. Apparent digestibility was improved by increased DCAD supplementation. Plasma leptin concentration was higher with DCAD supplementation.

**Conclusion:**

When feeding goats with DCAD-39 under tropical conditions, an increase in DMI was associated with improved apparent digestibility of nutrients, ruminal fermentation and microbial protein synthesis. An increase in plasma leptin concentration could not explain the effect of high DCAD on DMI.

## INTRODUCTION

Eating behavior is the main behavior contributing to mammary gland function in dairy animals [1]. Under high ambient temperature (HTa), it has been shown that decreased eating not only affects milk yield but also alters gastrointestinal and endocrine functions [1–3]. Negative effects of HTa on ruminal functions, such as lower ruminal pH, microbial activities and fermentation characteristics, have been well reported [2,3]. In dairy goats, leptin as an adiposity hormone has been shown to be involved in satiation [4]. Plasma leptin concentration increased in heat-stressed dairy goats [5]. A similar effect of HTa on plasma leptin in dairy cows has been reported as well [6,7].

It is known that potassium (K) and sodium (Na) requirements increase with HTa. Dairy cows increased dry matter intake (DMI) during K supplementation [8]. Heat-stressed cows increased DMI when dietary cation and anion difference (DCAD) level increased from 12 to 46 mEq/100 g dry matter (DM) [8,9]. The effect of high DCAD on DMI depended on the levels of DCAD and the stage of lactation in dairy cows [10,11]. Many previous experiments showed that the effects of high DCAD diet on DMI apparently derived in part from changes in ruminal function [12–14]. Our previous data demonstrated, as well, that high DCAD increased DMI in dairy goats [15,16]. However, the mechanisms by which high DCAD influences DMI in lactating dairy goats under HTa were not fully understood.

The present experiment was performed to investigate the possible mechanisms by which high DCAD diet affects DMI in lactating goats. We hypothesized that the effect of high DCAD on DMI is in part associated with alterations in ruminal function or partially mediated via plasma leptin concentration.

## MATERIALS AND METHODS

### Animals and management

The experiment was carried out at Nakornpathom training farm, Nakornpathom province, Thailand. The average temperatures during the experiment at 0700 h, 1300 h and 1900 h were 27.64°C±1.45°C, 35.28°C±0.50°C and 30.35°C±0.26°C, respectively. Relative humidities at 0700 h, 1300 h and 1900 h were 71.29%±2.92%, 50.60%±1.06% and 65.37%±0.41%, respectively. Values of the temperature and humidity index, calculated according to NRC’s formula [17] from the above information, were 78.12±2.13, 85.48±0.47, and 81.33±0.39, respectively. The procedures of this experiment were performed according to the guidelines for the use of animals from the National Research Council of Thailand and were approved by the Animals Care and Use Committee, Faculty of Veterinary Science, Chulalongkorn University (#1531074).

Ten crossbred Saanen goats that were in late pregnancy (3 to 4 years old) and with an average body weight (BW) 34.48 ±1.42 kg were selected and used in this experiment. For adaptation, from one month before parturition all animals were kept in individual metabolic cages with plastic floors (2×1 m). A week after parturition (PP-1), animals were randomly divided into two groups based on BW and milk yield, with five animals in each group, and studied for 7 weeks (2nd to 8th weeks of postpartum, PP-2 to PP-8). They were offered two experimental rations of either control (DCAD, 22.81 mEq/100 g DM; DCAD-23) or high DCAD (DCAD, 39.08 mEq/100 g DM; DCAD-39). The rations contained 44% corn silage and 56% concentrate and were formulated as total mixed ration (TMR) according to NRC recommendation [18]. NaHCO_3_ and K_2_CO_3_ were added to the rations depending on the assigned levels of DCAD. Ingredients and chemical compositions of the rations are described in Table 1. Dairy goats received TMR *ad libitum*, twice daily at 0700 h and 1400 h. Goats had free access to water. After parturition, all the goats were weighed before morning feeding, once per week throughout the experiment.

### Data collection and feed analysis

Feed intake (FI) and water intake (WI) were recorded daily and then calculated separately for morning (from 0700 h to 1300 h), afternoon (from 1300 h to 1900 h), night (from 1900 h to 0700 h) and the whole day (24 h). Feed and refusal samples were collected every day throughout the experiment and divided into two parts; one half was immediately dried in an oven at 105°C until constant weight to determine DM, and the remaining samples were kept frozen at −20°C until chemical analysis. At the end of the experiment all the feed samples were thawed and mixed thoroughly, and subsamples were dried at 65°C overnight (approximately 12 h) for nitrogen and ash analysis according to AOAC [19] and to determine neutral detergent fiber (NDF) and acid detergent fiber (ADF) using the procedure developed by Van Soest et al [20]. Sodium and potassium were measured by atomic absorption spectrophotometer (Thermo iCE 3000 series, Thermo Fisher Scientific, Cambridge, UK), chloride (Cl) was determined by colorimetric titration and sulfate (SO_4_^2−^) was measured by spectrophotometer (UV-VIS 1800 Shimadzu, Kyoto, Japan).

### Rumen collection and volatile fatty acids analysis

Rumen fluid samples were collected from each dairy goat once on day 7 of PP-8 using a stomach tube connected to a syringe. To avoid excessive saliva contamination, the double tubes technique was used for rumen fluid collection. The outer rubber tube (i.d. = 2.2 cm) was fixed to the mouth gag. The inner rubber tube (o.d. = 1.0 cm) was the collecting tube (110 cm) that passed into the ruminal cavity. Approximately 20 mL of fluid samples were taken at 2.5 h after morning feeding. The pH was immediately determined with a pH meter (pH221, Lutron, Taipei, Taiwan). After that, the ruminal fluid samples were filtered through two layers of cheese-cloth and 1 mL 6 N HCl was added for preservation. Then, samples were frozen at −20°C for later analysis of osmolality, volatile fatty acids (VFAs) and NH_3_-N. The ruminal fluid osmolality was measured with an osmometer (Osmometer 3D3; Advanced Instruments Inc., Boston, MA, USA). The VFAs were prepared and analyzed as described by Thammacharoen et al [21]. NH_3_-N was determined with a salicylate-hypochlorite method [22].

### Apparent digestibility

The digestibility was measured using total fecal collection technique. The feces of each goat were collected daily and were mixed t at PP-8 (7 days). The subsamples from each animal were kept under −20°C for later analysis. The fecal samples were analyzed for DM, organic matter (OM), crude protein (CP), ADF, and NDF levels as previously described. Calculation of the percentage of apparent digestibility was done by dividing the difference between the nutrient amounts in feed and fecal excretions by the amount of nutrient in feed.

### Urine collection and analysis of allantoin excretion

Total urine was collected and measured in the same week as fecal collection by using plastic containers with 10% sulfuric acid solution added to prevent nitrogen loss (15 mL H_2_SO_4_ 10% in 90 mL urine), and the final pH of urine was kept below 3. The total urine from each day was then sampled (30 mL), kept under −20°C and pooled at the end of experiment to be analyzed for nitrogen by the Kjeldahl method [18] and for allantoin excretion by a colorimetric method according to Chen and Gomes [23]. Nitrogen retention was calculated from the difference between nitrogen input (from FI) and nitrogen output (from the total nitrogen in feces, urine, and milk).

### Determination of plasma leptin concentration

On day 27 and day 55 postpartum (PP-4 and PP-8), blood samples were collected at 1600 h for analysis of plasma leptin concentration. The samples were obtained from the jugular vein, placed in lithium heparin tubes, kept in crushed ice and then centrifuged at 3,000 rpm for 10 minutes. The plasma samples were stored at −20°C until analysis. Plasma leptin concentration was determined using an enzyme-linked immunosorbent assay kit specific for multispecies hormones (MBS018743, MyBioSource, San Diego, CA, USA). The sensitivity of this kit was 1 ng/mL. The intra-assay variation for this measurement was 7.82%.

### Statistical analysis

The data were presented as the mean±standard error of the mean. The data for plasma leptin were analyzed with repeated two-way analysis of variance (ANOVA). Significance of main effects was determined by Bonferroni posttest. The data for FI, WI, ruminal parameters, and nutrient digestibility were averaged and compared using an unpaired T-test. Significance was declared at p<0.05. A tendency was declared at 0.05< p<0.10.

## RESULTS

### DCAD-39 effect on dry matter intake, water intake and digestibility

**Figure 1:**
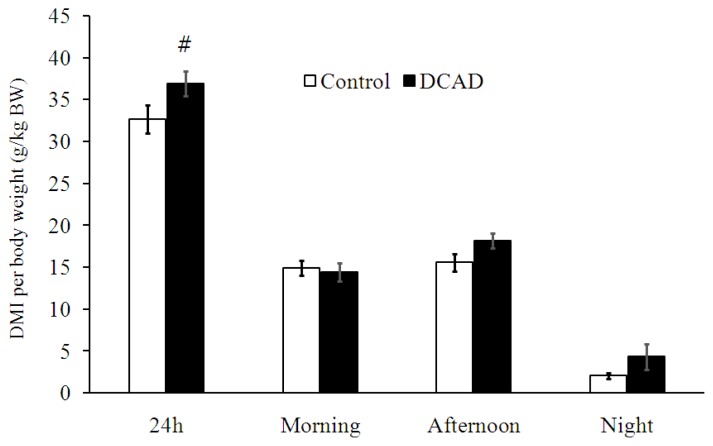
Effects of dietary cation and anion difference (DCAD) on the averaged 24-h dry matter intake per body weight (DMI/BW), morning DMI/BW from 0700 h to 1300 h, afternoon DMI/BW from 1300 to 1900 h, night-time DMI/BW from 1900 to 0700 h. The DCAD levels of the DCAD-23 and DCAD-39 groups were 22.8 and 39.1 mEq/100 g DM, respectively. Pound sign indicates the tendency of difference, ^#^ 0.05<p<0.1.

When dry matter intake per body weight (DMI/BW) was averaged throughout the experiment, there were no differences in DMI/BW between DCAD-23 and DCAD-39 groups during morning, afternoon, and night time feeding (p>0.05, Figure 1). However, goats in the DCAD-39 group tended to consume more total daily DMI/BW than did those in DCAD-23 (p = 0.095, Figure 1). When DMI/BW was averaged weekly, significantly higher DMI/BW at PP-8 was apparent in the DCAD-39 group (p<0.05), while the ratio of DMI:WI was not significantly different (p>0.05, Table 2). Moreover, the analysis of nutrient intake at PP-8 revealed that CP intake from the DCAD-39 group was higher than that from the DCAD-23 group (p<0.05; Table 2). Nutrients digestibility measured at PP-8 for DM, OM, CP, NDF, and ADF in the DCAD-39 group were higher than in the DCAD-23 group (p<0.05, Table 2).

**Figure 2:**
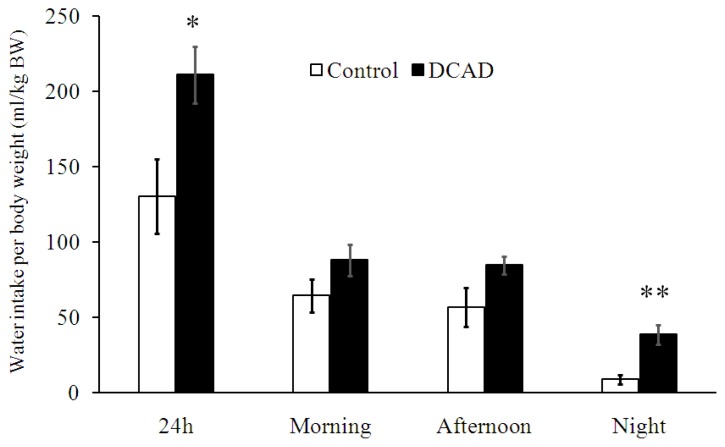
Effects of dietary cation and anion difference (DCAD) on the average 24 h water intake (WI), morning WI from 0700 to 1300 h, afternoon WI from 1300 to 1900 h; night-time WI from 1900 to 0700 h. The DCAD levels from DCAD-23 and DCAD-39 groups were 22.8 and 39.1 mEq/100 g DM, respectively. Asterisks indicate significant differences within each period, * p<0.05; ** p<0.01.

There was an effect of DCAD supplementation on total daily and night time WI; animals in DCAD-39 group drank more water than did those in the DCAD-23 group (p<0.05, Figure 2). However, WI was similar between the two groups (p>0.05, Figure 2) during morning and afternoon feedings.

### DCAD-39 influenced ruminal fermentation patterns and nitrogen balance

Ruminal fluid pH was higher for goats in the DCAD-39 group than for those in the DCAD-23 group (p<0.05, Table 3). The average NH_3_-N concentration was not affected by DCAD level (p>0.05). Total VFA concentration, propionate molar proportion and average ratio of acetate to propionate were not affected by DCAD supplementation (p>0.05). In contrast, acetate molar proportion was greater, and butyrate molar proportion was lower, in the DCAD-39 group than in the DCAD-23 group (p<0.05). Ruminal microbial supply to the lower gut was indirectly evaluated from allantoin excretions at PP-8 (Table 4). Urine output and urinary allantoin excretion were greater in goats fed with DCAD-39 than in those fed with DCAD-23 (p<0.05).

The apparent nitrogen balance was calculated from nitrogen input and output (Table 4). There were no significant differences in nitrogen intake or excretion in urine, feces or milk (p>0.05). As a result, nitrogen balance was similar between the two groups (p>0.05).

### Plasma leptin concentration

**Figure 3:**
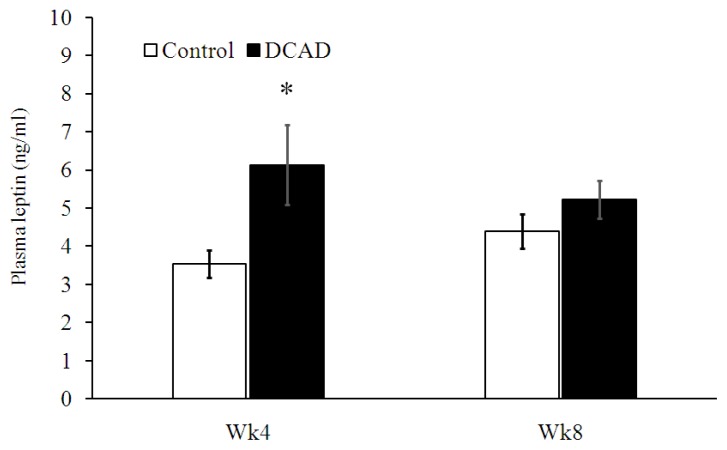
Effect of dietary cation and anion difference (DCAD) on plasma leptin concentration in lactating dairy goats at 4th and 8th weeks postpartum. The DCAD levels from DCAD-23 and DCAD-39 groups were 22.8 and 39.1 mEq/100 g DM, respectively. Asterisks indicate the significant differences within each period, * p<0.05.

The average plasma leptin concentrations of goats in DCAD-23 and DCAD-39 groups were 4.0±0.3 and 5.7±0.6 ng/mL, respectively (Figure 3). Specifically, plasma leptin was significantly higher in DCAD-39 goats than in DCAD-23 goats at PP-4 (p<0.05).

## DISCUSSION

In the present study, the DCAD-39 effect on WI was apparent when comparing it with the effect on DMI. DCAD-39 increased WI and urine output and tended to increase DMI. Ruminal fermentation patterns and nutrient digestibility changed in association with drinking and eating responses. However, the DCAD effect on plasma leptin could not explain the effects of DCAD on eating.

Under HTa, the mechanisms by which DCAD-39 diet increased WI is straightforward, since DCAD-39 caused higher plasma cationic difference (mainly from sodium ions) and thereby directly stimulated the thirst center [15]. The accumulation of water from night-time drinking may be a limiting factor for DMI. The increase in DMI/BW was associated with improvements in ruminal fermentation patterns (Tables 2, 3) including nutrient digestibility, ruminal fluid pH, VFA and microorganism activities, rather than changes in plasma leptin. Since the DCAD-39 diet increased WI significantly, the first question that should be answered is whether water or DCAD is the principal factor influencing ruminal fermentation patterns. However, the comparable values of DMI:WI (Table 1) and ruminal fluid osmolality (Table 2) reported in the present experiment suggest that increases in WI could not dilute the ruminal content. Hence, DCAD-39 diet may have been the major factor influencing ruminal fermentation patterns.

We showed in the present results that total apparent digestibilities of DM, OM, CP, NDF, and ADF increased in response to DCAD-39 diet. The effects of varying DCAD on nutrient digestibility were reported by Delaquis and Block [10]. They found that ADF and NDF digestibilities did not differ with high DCAD in dry and lactating cows, but DM digestibility was slightly higher in lactating cows. Stokes and Bull [24] found that supplementation with sodium bicarbonate as a cationic salt for high DCAD improved DM, OM, and ADF digestibilities when dairy cows were fed a corn silage based diet rather than alfalfa hay. Similarly, DM, OM, and NDF digestibilities increased with KHCO_3_ supplementation in dairy cows [14]. These findings were similar to those of the present experiment in that the dairy goats in our study were fed with corn silage as roughage source. The increase in nutrient digestibility in the present study was associated with the positive effect of DCAD-39 diet on rumen fermentation patterns. The ruminal fluid pH at 2.5 h after morning feeding increased with higher DCAD. The higher ruminal pH resulted mainly from the bicarbonate buffer added to prepare the DCAD-39 ration. In the present experiment, the concentrations of acetate and butyrate were affected by DCAD supplementation. Dairy goats fed with DCAD-39 had higher ruminal acetate levels than those fed with DCAD-23. However, butyrate molar proportion was lower in goat fed DCAD-39 diet than in those fed with DCAD-23. The levels of NH_3_-N, total VFA, propionate molar proportion and average ratio of acetate to propionate were similar between the two groups. The finding regarding ruminal function in this study were consistent with those of previous investigations; dairy cows fed with DCAD of 16 to 53.5 mEq/100 g DM exhibited increased ruminal pH and acetate concentrations and tended to show reduced butyrate levels [25]. In contrast, ruminal pH, ruminal ammonia nitrogen, acetate levels and acetate: propionate ratios were greater in buffalo bulls fed with medium and high DCAD [26] than in those fed anion diets or low DCAD (110 mEq/kg DM) diets. The effect of DCAD level on concentrations of VFA, acetate, propionate and butyrate in this study may relate to the microbial activity of DCAD-39.

Dairy goats in the DCAD-39 group had higher urinary allantoin excretion than did those in the DCAD-23 group. Higher allantoin excretion apparently resulted from the increased in ruminal pH caused by DCAD-39 diet. Elevated water consumption may have contributed to higher allantoin excretion in part via increases in rumen liquid fractional passage rate and in microbial growth rate, as reported by Dijkstra et al [27]. Dairy cows in mid-lactation fed with high potassium bicarbonate (cationic salt) exhibited higher allantoin excretion than did those fed with low potassium bicarbonate [14]. Although our present experiment did not quantify the microbial protein flowing to the small intestine, we do think that an extra nitrogen pool would be higher in the DCAD-39 group [27]. In addition, it has been shown that DCAD-39 apparently improves ruminal nitrogen utilization as indicated by lower blood urea nitrogen [13]. Plasma concentration of branched chain amino acids and the ratio of essential amino acids:total amino acids were higher in dairy cows fed with a DCAD of 47 mEq/100 g DM during early lactation than in those fed with DCAD of 22 mEq/100 g DM [28]. An increase in allantoin excretion suggested that DCAD-39 improves microbial protein synthesis. When analysis was performed on the nitrogen balance including nitrogen intake and excretion in urine, milk and feces, it was determined that DCAD-39 diet did not change this balance. Similar findings were found by Delaquis and Block [10], when dairy cows were fed with high DCAD. However, nitrogen intake and retention were higher in buffalos fed with medium and with high DCAD than in those fed with low or negative DCAD [26]. If we consider ruminal nitrogen balance in term of dietary and microbial pools, the comparable feces N excretion and the apparently higher microbial protein supply based on allantoin excretion implies that nitrogen balance might be more favorable in the DCAD-39 group. Taken together, the present findings of urinary allantoin excretion and nitrogen balance suggest that DCAD-39 diet improves rumen nitrogen utilization by changing microbial activity. To test the latter hypothesis, the effect of DCAD-39 on both dietary and microbial ruminal nitrogen digestibilities should be investigated.

In addition to nutrient digestibility, ruminal fermentation patterns and microbial activity, there is a hormonal factor related to FI that we investigated to determine whether DCAD-39 diet would change FI in part by this endocrine mechanism. Previously, we demonstrated that leptin may be related to growth hormone induced decreased FI in dairy goats [6]. In the present experiment, plasma leptin concentrations were significantly higher in the DCAD-39 group, mainly from PP-4. In principle, leptin acts on hypothalamic neurons to inhibit food intake and increase energy expenditure, leading to reduced BW [29]. The higher plasma leptin in the DCAD-39 group reported in this experiment could not explain the effect of DCAD-39 diet on FI for two reasons. First, higher plasma leptin should decrease rather than increase DMI in animals fed DCAD-39 diet [29]. Second, the significant effects of DCAD level on plasma leptin (PP-4) and DMI (PP-8) were uncoupled.

## CONCLUSION

We present the associated gastrointestinal mechanisms that contribute to the effect of the DCAD-39 diet on FI under HTa. The DCAD-39 diet increased ruminal fermentation patterns in part by an increase in microbial activity. The gastrointestinal responses of goats fed the DCAD-39 in the present experiment apparently resulted from the positive DCAD rather than its effect on drinking behavior. The improvements in ruminal function and production facilitate the eating-behavior effect of the DCAD-39 diet. Finally, increased plasma leptin concentration in dairy goats fed DCAD-39 diet were not associated with the diet’s effect on DMI.

## Figures and Tables

**Table 1:** Feed ingredients and chemical compositions of experimental diets (DM basis) differing in dietary cation-anion difference

Items	DCAD-23	DCAD-39
Ingredients (%)		
Corn silage	44.00	44.00
Cassava	3.26	3.26
Soybean meal	19.62	19.62
Molasses	3.69	3.69
Corn meal	25.86	24.42
Rice bran	2.25	2.25
Limestone	0.90	0.90
NaHCO_3_	0.14	0.62
K_2_CO_3_	0.28	1.24
Analyzed nutrient composition (%)		
DM	35.69	35.49
CP	16.68	16.84
Ash	6.36	7.24
OM	93.64	92.76
ADF	26.12	25.16
NDF	51.61	50.03
Na (meq/100 g DM)	4.48	7.00
K (meq/100 g DM)	36.78	47.10
Cl (meq/100 g DM)	10.57	5.71
S (meq/100 g DM)	7.87	9.31
Calculated nutrient composition (%)		
Ca1)	0.52	0.54
P1)	0.36	0.49
NE_L_1) (Mcal/kg DM)	1.66	1.67
DCAD2) (mEq/100 g DM)	22.81	39.08

DM, dry matter; DCAD, dietary cation-anion difference; CP, crude protein; OM, organic matter; ADF, acid detergent fiber; NDF, neutral detergent fiber; Na, sodium, K, potassium; Cl, chloride; S, sulphure; Ca, calcium; P, phosphorus; NE_L_, net energy for lactation.

1) Calculated according to NRC (1981).

2) DCAD, in miliequivalents of (Na+K)−(S+Cl)/100 g of DM.

**Table 2:** Effects of dietary cation and anion difference on dry matter intake, the ratio of dry matter intake and water intake, nutrient intake and apparent digestibility at 8th weeks postpartum in dairy goats

Items	DCAD-231)	DCAD-391)	p-value
DMI (g/kg BW)	32.75±1.85	38.11±1.21	0.04
DMI:WI ratio	1.03±0.19	0.60±0.07	0.07
Nutrient intake (g/kg BW/d)			
Organic matter	30.67±1.73	35.35±1.12	0.053
Crude protein	5.46±0.31	6.42±0.20	0.03
Neutral detergent fiber	16.90±0.95	19.07±0.61	0.09
Acid detergent fiber	8.56±0.48	9.59±0.30	0.11
Apparent digestibility (%)			
Dry matter	74.40±0.90	78.75±0.83	0.001
Organic matter	68.05±1.48	74.86±1.26	0.001
Crude protein	73.03±1.24	78.08±1.47	0.02
Neutral detergent fiber	59.15±2.23	67.85±1.44	0.01
Acid detergent fiber	48.62±4.05	59.24±1.79	0.02

DCAD, dietary cation and anion difference; DMI, dry matter intake; BW, body weight; WI, water intake; DM, dry matter.

1) DCAD-23, 22.81 mEq/100 g DM; DCAD-39, 39.08 mEq/100 g DM.

**Table 3:** Effects of dietary cation and anion difference on ruminal fermentation characteristics in dairy goats

Items	DCAD-231)	DCAD-391)	p-value
Ruminal fluid pH	6.54±0.05	6.68±0.01	0.01
NH_3_-N (mg/dL)	18.10±2.35	17.03±1.84	0.71
Total VFA (mmol/L)	64.25±4.85	63.97±2.33	0.96
VFA (mol/100 mol)			
Acetate	54.81±1.05	59.25±1.54	0.04
Propionate	34.85±1.25	33.00±1.34	0.40
Butyrate	10.34±0.55	7.75±0.45	0.01
Acetate:propionate ratio	1.58±0.08	1.82±0.15	0.22
Osmolality (mOsm/kg)	241.20±25.89	223.20±11.80	0.48

DCAD, dietary cation and anion difference; VFA, volatile fatty acid; DM, dry matter.

1) DCAD-23, 22.81 mEq/100 g DM; DCAD-39, 39.08 mEq/100 g DM.

**Table 4:** Effects of dietary cation and anion difference on urinary allantoin excretion and nitrogen balance at 8th weeks postpartum in dairy goats

Items	DCAD-231)	DCAD-391)	p-value
Urine (kg/d)	2.04±0.52	3.95±0.30	0.013
Allantoin excretion (mg/BW/d)	13.41±2.65	22.75±3.01	0.05
Nitrogen intake (g/d)	30.86±2.60	34.89±1.92	0.25
Nitrogen excretion (g/d)			
Urine	8.73±0.76	9.78±0.76	0.34
Feces N	6.25±0.51	5.92±0.47	0.65
Milk	6.48±0.98	6.78±0.85	0.82
Nitrogen balance (g/d)	9.40±1.72	12.39±1.24	0.20

DCAD, dietary cation and anion difference; BW, body weight.

1) DCAD-23, 22.81 mEq/100 g DM; DCAD-39, 39.08 mEq/100 g DM.
